# Generation of an NG2 targeting bispecific antibody for the induction of T-cell immunity against melanoma

**DOI:** 10.1186/s12967-026-09000-5

**Published:** 2026-09-19

**Authors:** Nisha Prakash, Sebastian Hörner, Ilona Hagelstein, Josephine Keller, Kevin Wang, Gundram Jung, Helmut R. Salih, Martina S. Lutz

**Affiliations:** 1 Clinical Collaboration Unit Translational Immunology, Department of Internal Medicine, University Hospital Tuebingen, Tuebingen, Germany; 2 German Cancer Consortium (DKTK), Partner Site Tuebingen, A Partnership between DKFZ and University Hospital Tuebingen, Tuebingen, Germany; 3 Cluster of Excellence iFIT (EXC 2180) “Image-Guided and Functionally Instructed Tumor Therapies”, University of Tuebingen, Tuebingen, Germany; 4 Institute of Immunology, University of Tuebingen, Tuebingen, Germany

**Keywords:** Bispecific antibodies, NG2, CD3 affinity, Melanoma

## Abstract

**Background:**

Melanoma remains a highly aggressive malignancy, and many patients fail to achieve durable benefit despite immune checkpoint inhibitor (ICI) therapy. ICIs improve outcomes but lack tumor specificity and do not directly recruit T-cells to tumor cells resulting in substantial side effects, highlighting the need for targeted immunotherapies selectively toward melanoma. Neuron glial antigen-2 (NG2) represents an attractive antigen for T-cell redirecting strategies due to its largely tumor restricted expression.

**Methods:**

Here we report the generation and characterization of T-cell engaging NG2‑targeting bispecific antibodies. (bsAbs). To finetune CD3 affinity, we generated two constructs with differing CD3-affinities. NG2xCD3*high* contains a UCHT-1-based CD3-binder with high affinity, whereas NG2xCD3*low* carries a UCHT‑1 variant with approximately 100‑fold reduced CD3 affinity. Both constructs were functionally assessed in NG2-high and NG2-low melanoma cell lines covering several key aspects of T-cell mediated immune response.

**Results:**

In cultures with NG2-expressing melanoma cells, both constructs mediated immune-synapse formation, T-cell activation, proliferation, and strictly NG2-dependent tumor cell killing. Both NG2xCD3*high* and NG2xCD3*low* supported differentiation into memory T‑cell subsets, but NG2xCD3*low* induced less cytokine secretion and diminished functional activity, particularly against NG2 lower expressing targets. NG2xCD3*high* maintained robust cytokine production and sustained cytotoxicity across both NG2‑high and NG2‑low tumor models.

**Conclusion:**

Our data identify NG2 as a promising target for T-cell-redirecting therapy in melanoma and demonstrate that, within the IgG-scFv format, high CD3 affinity is required to achieve potent effector function across differing NG2-expression levels. NG2xCD3*high* was thus identified as lead candidate for further development in NG2-expressing malignancies.

**Supplementary Information:**

The online version contains supplementary material available at https://doi.org/10.1186/s12967-026-09000-5.

## Background

Melanoma is a heterogenous and aggressive form of skin cancer with high metastatic potential [1, 2]. It arises from melanocytes located at multiple anatomical sites and therefore comprises biologically and clinically distinct disease entities. Clinically, melanomas are commonly categorised as cutaneous (including non-acral and acral lesions derived from epidermal melanocytes), mucosal (arising from melanocytes within mucous membranes), and uveal/ocular (originating from melanocytes in the uveal tract), with additional special-site and unknown primary forms recognized in contemporary classifications [3–5]. Cutaneous melanoma is the predominant subtype, accounting for more than 90% of all cases [6, 7]. Globally, the incidence of cutaneous melanoma continues to rise, and projections from the International Agency for Research on Cancer (IARC) indicate that the number of new cases will increase by more than 50% to roughly 510,000 and deaths to about 96,000 by 2040 [8].

Advanced melanoma has historically been associated with poor prognosis and resistance to conventional treatments such as surgery, chemotherapy and radiotherapy [9]. Over the past decade, the therapeutic landscape has been transformed by immune checkpoint inhibitors (ICIs) and small-molecule targeted therapies, particularly BRAF and MEK inhibitors [10–13]. However, treatment efficacy is limited by primary and acquired resistance as well as immune-related adverse events. Approximately 40–50% of patients fail to achieve durable benefit with current regimens, underlining the need for additional treatment strategies requiring new melanoma-associated targets that can complement existing immunotherapies [14, 15].

NG2 (neuron glial antigen-2), the rat homolog of human chondroitin sulphate proteoglycan 4 (CSPG4), represents a highly attractive melanoma‑associated target [16, 17]. NG2/CSPG4 (hereafter termed NG2) is a type I transmembrane proteoglycan with a large, heavily glycosylated extracellular domain and comparatively short transmembrane and cytoplasmic regions. While its physiological function remains incompletely understood, NG2 is highly and frequently expressed on cutaneous and uveal melanoma [18]. It was initially identified in melanoma and has been shown to promote tumor cell proliferation, survival and motility, in part by orchestrating interactions between melanoma cells and their microenvironment [19].

Several NG2-directed immunotherapeutic strategies have been explored, including monoclonal antibodies (mAbs), antibody-drug conjugates, chimeric antigen receptor (CAR) T- cells and bispecific T-cell engagers [20–22]. CD3-bispecific antibodies (bsAbs) directed against NG2 represent attractive off-the-shelf compounds to recruit T-cells for elimination of NG2 expressing melanoma cells. The choice of given tumor antigens is critical to maximize on-target activity while minimizing off-tumor toxicity. NG2 fulfils these requirements owing to its restricted expression in normal tissues, its high and consistent expression on melanoma cells, and its presence on tumor-associated pericytes during angiogenesis, enabling simultaneous targeting of malignant cells and supportive vasculature [23, 24].

Building upon our previous work on CD3-bsAbs, including FLT3xCD3, PSMAxCD3, and B7-H3xCD3 molecules that have progressed to clinical evaluation (NCT05143996, NCT04104607, NCT04496674, NCT05999396), we here preclinically evaluated NG2xCD3 bsAbs and systematically compared different CD3 affinities [25–29]. This approach acknowledges that any given tumor antigen requires its own optimal CD3 binder to achieve balanced activity, controlled cytokine release, and an accordingly appropriate therapeutic window.

## Methods

### TCGA/GEPIA analysis of NG2

Relative NG2 mRNA expression in tumor and normal tissues was analyzed using RNA‑sequencing data from The Cancer Genome Atlas (TCGA) project, with corresponding normal tissue expression obtained from the Genotype-Tissue Expression (GTEx) resource within the TCGA/GTEx framework. For cutaneous melanoma, NG2 expression in skin cutaneous melanoma (SKCM) samples versus healthy tissue samples were visualized as box plots using the Gene Expression Profiling Interactive Analysis (GEPIA) web server, as described previously [30–32]. In addition, NG2 expression datasets for glioblastoma (GBM), head and neck squamous cell carcinoma (HNSC), kidney renal clear cell carcinoma (KIRC), lower‑grade glioma (LGG), pancreatic adenocarcinoma (PAAD), and thymoma (THYM) were retrieved from TCGA for further downstream analyses.

### Generation and purification of NG2 bsAbs

For generation of humanized NG2xCD3 bsAbs, the murine anti‑NG2 mAb 9.2.27 was used. The variable heavy (VH) and variable light (VL) sequences of 9.2.27 were aligned to human germline immunoglobulin gene databases using IgBLAST (http://www.ncbi.nlm.nih.gov/igblast/) and IMGT/V‑QUEST (http://www.imgt.org/IMGT_vquest/input), and Kabat‑defined complementarity-determining region (CDR) residues were grafted onto selected human *κ* light- and heavy-chain framework sequences. The T20 humanness score, as defined by Gao et al. [33] was calculated for all VH and VL variants to quantify their similarity to human antibody repertoires. Variable domains derived from 9.2.27 were combined in different VH/VL pairings and cloned into a human IgG1κ‑based IgG‑sc bispecific format. This construct incorporates LALA (L234A/L235A) for Fc-silencing and YTE mutations (M25Y/S254T/T256E) to extended serum half-life, along with a C‑terminal scFv conferring CD3 specificity, resulting in eight NG2xCD3 bsAb variants. In parallel, variable domains from the non‑targeting mouse IgG1κ antibody MOPC‑21 were cloned into the same IgG‑sc architecture to generate matched isotype control molecules. The lead NG2‑binding VH/VL combination was selected based on production yield, T20 humanness scores, and suitability for downstream functional characterization. The constructs were produced in ExpiCHO cells (Gibco, Carlsbad, CA) and purified from the cell culture supernatant via affinity chromatography using MabSelect columns (Cytiva, Marlborough, MA, USA). Preparative and analytical size-exclusion chromatography was carried out on Superdex200 HiLoad and 16/60 Increase 10/300 GL columns (Cytiva).

### Cell lines and PBMCs

Human melanoma cell lines A375, SK-MEL-28, SK-MEL-2, MEL-JUSO and UACC-257, of which SK-MEL-28, SK-MEL-2, and UACC-257 are part of the NCI-60 human tumor cell line panel (National Cancer Institute, Bethesda, MD), were cultured in Gibco DMEM (Thermo Fisher Scientific, Waltham, MA) supplemented with 10% FBS (Capricorn, Ebsdorfergrund, Germany) and standard antibiotics. Cell cultures were routinely screened for mycoplasma contamination at three‑month intervals, and only mycoplasma‑negative cells were used for experiments. Peripheral blood mononuclear cells (PBMCs) from healthy donors were isolated by density‑gradient centrifugation using Biocoll (Biochrom, Berlin, Germany).

### Flow cytometry-based assays

For analysis of NG2 surface expression, cells were stained with the parental murine anti‑NG2 antibody 9.2.27 or the corresponding mouse IgG isotype control (clone MOPC‑21) at a concentration of 10 µg/mL. The primary antibodies were detected using PE‑conjugated goat anti‑mouse IgG (Jackson ImmunoResearch West Grove, PA). NG2 surface density was quantified by flow cytometry using the murine 9.2.27 mAb and QIFIKIT calibration beads (Dako, Hamburg, Germany) according to the manufacturer’s protocol.

For binding titration of the humanized NG2xCD3 bsAbs, bound antibody was detected with a PE‑conjugated donkey anti‑human IgG secondary antibody, and EC_50_ values were determined using GraphPad Prism 10 (GraphPad Software). T-cell activation and tumor cell depletion were assessed in flow cytometry-based assays in which PBMCs were cocultured with A375 or SK-MEL-2 target cells at defined effector‑to‑target (E: T) ratios in the presence or absence of bsAbs. T-cells were identified using fluorochrome-conjugated antibodies to CD4 and CD8. T-cell activation and memory differentiation were assessed using CD69, CD25, CD45RO, and CD62L (all BioLegend, San Diego, CA, USA). For analysis of inhibitory receptor expression following prolonged stimulation, T cells were additionally stained for PD-1, TIM-3, and LAG-3 (all BioLegend, San Diego, CA, USA). Tumor cells were detected using a B7-H3 antibody or were labeled with CellTrace™ Violet (Thermo Fisher Scientific, Waltham, MA, USA). Absolute cell numbers were calculated using a fixed number of BD CompBeads (BD Biosciences San Diego CA), and dead cells were excluded by 7‑AAD staining (BioLegend) or Fixable Aqua live/dead staining (Thermo Fischer Scientific). To investigate the potential synergistic effects of NG2xCD3 bsAbs and PD-1 blockade, PBMCs were co-cultured with A375-PD-L1 target cells at an effector-to-target (E: T) ratio of 5:1 in the presence of NG2xCD3 bsAbs (1 µg/ml) with or without pembrolizumab (Keytruda^®^, Merck Sharp & Dohme, Rahway, NJ, USA; 25 µg/ml). For short-term assays, tumor cell lysis was quantified by flow cytometry after 72 h. For rechallenge experiments, fresh target cells together with fresh NG2xCD3 bsAbs (1 µg/ml) and pembrolizumab (25 µg/ml) were added after 72 h of co-culture, and tumor cell lysis was determined by flow cytometry after a total culture period of 6 days. For cytokine secretion analyses, cell culture supernatants were collected after 24 h and analyzed using the Th1 LEGENDplex multiplex bead‑based assays (BioLegend, catalogue no: 741036) according to the manufacturer’s instructions. All samples were acquired on a BD FACSCanto™ II or BD LSRFortessa™ flow cytometer (BD Biosciences, San Jose, CA, USA), and data were analyzed using FlowJo software (FlowJo LLC, Ashland, OR, USA).

### Cell viability assays

Melanoma cells (10,000 cells/well) were cultured for 72 h in the presence or absence of the indicated constructs (1 µg/mL). Cell viability and metabolic activity were subsequently assessed using WST reagent (Roche) or the CellTiter-Glo^®^ Luminescent Cell Viability Assay (Promega, Madison, WI) according to the manufacturer’s instructions.

### Image stream

A375 and SK‑MEL‑2 cells were cocultured with PBMCs from healthy donors at an E: T ratio of 2.5:1 in the presence or absence of the two NG2xCD3 bsAbs for 1.5 h. After incubation, cells were stained with a FITC‑labeled anti‑human Fc-specific antibody (Jackson ImmunoResearch) to detect cell‑bound NG2xCD3, followed by surface staining with CD4‑PECy7 (BioLegend), CD8‑BV605 (BioLegend), and B7‑H3‑PE (BioLegend) and then fixed in 1.5% paraformaldehyde (PFA) in PBS for 15 min at room temperature. Cells were washed with PBS and permeabilized with PBS containing 0.1% Tween‑20 (PBS‑T) for 20 min at room temperature, after which DAPI (0.1 µg/mL; Invitrogen,) and Phalloidin‑AF647 (1:500; Thermo Fisher Scientific) were added to stain nuclei and F‑actin, respectively. A minimum of 100,000 events were acquired at 40x magnification on an Amnis ImageStream MkII imaging flow cytometer (Cytek Biosciences, San Diego, CA), and data were analyzed using IDEAS image analysis software. Samples were gated on binucleated aggregates, and organized immunological synapses were defined by quantifying colocalization of tumor and T-cell images within regions of polarized phalloidin signal.

### T-cell proliferation assay

For the ³H-thymidine uptake assay, irradiated target cells (2 × 10^4^) were incubated with healthy donor PBMCs (2 × 10^5^) seeded in triplicates in 96 well plates in the presence or absence of the indicated bsAbs. After 48 h, cells were pulsed with ³H-methyl-thymidine (0.5 µCi/well) for 20 h and harvested on filter mats. Incorporated radioactivity was determined by liquid scintillation counting in a 2450 MicroBeta microplate counter (PerkinElmer, Waltham, MA).

For flow cytometric-based proliferation assay, PBMCs were stained with 3 µM CellTrace™ Violet dye (Thermo Fisher Scientific) and incubated up to 4 days with the indicated unlabeled tumor cell lines. On day 3, fresh tumor cells and a new dilution of the bsAbs were added to the cultures, and proliferation was analyzed on day 4 as mentioned above.

### Cytotoxic assays

Real‑time cytotoxicity was assessed using the xCELLigence RTCA system (Roche Applied Science, Penzberg, Germany), with impedance measurements acquired every 15 min over 72 h period. Data are presented as means ± standard deviation (SD) of technical triplicates.

For long‑term live‑cell imaging, an IncuCyte S3 Live‑Cell Analysis System (Essen BioScience, Sartorius, Göttingen, Germany) was used to monitor cytotoxicity over up to 14 days. Nuclight Red‑labeled A375 or SK‑MEL‑2 cells, generated via transduction with Incucyte Nuclight Red lentivirus (Sartorius), were seeded in 96‑well plates and cocultured with effector cells at defined E: T ratios in the presence of bsAbs at the indicated concentrations. Phase‑contrast and fluorescent images were acquired every 6 h at 20x magnification, and on day 1 and day 7 fresh labeled target cells and antibodies were added. Red fluorescent Nuclight‑positive tumor cells were quantified and normalized either to the initial tumor cell count at t = 0 h or to the count at t = 168 h (day 7), as specified in the corresponding experiments.

### Statistics

All statistical analyses were carried out in GraphPad Prism 10. Data are shown as mean ± standard deviation (SD). Depending on data distribution and variance, group comparisons were performed using either one-way ANOVA with Dunnett’s test or the appropriate nonparametric tests, including the Wilcoxon matched-pairs test or the Mann–Whitney U test. Statistical significance was defined as *p* < 0.05.

## Results

### NG2 expression in melanoma and generation of NG2xCD3 bsAb

**Fig. 1:**
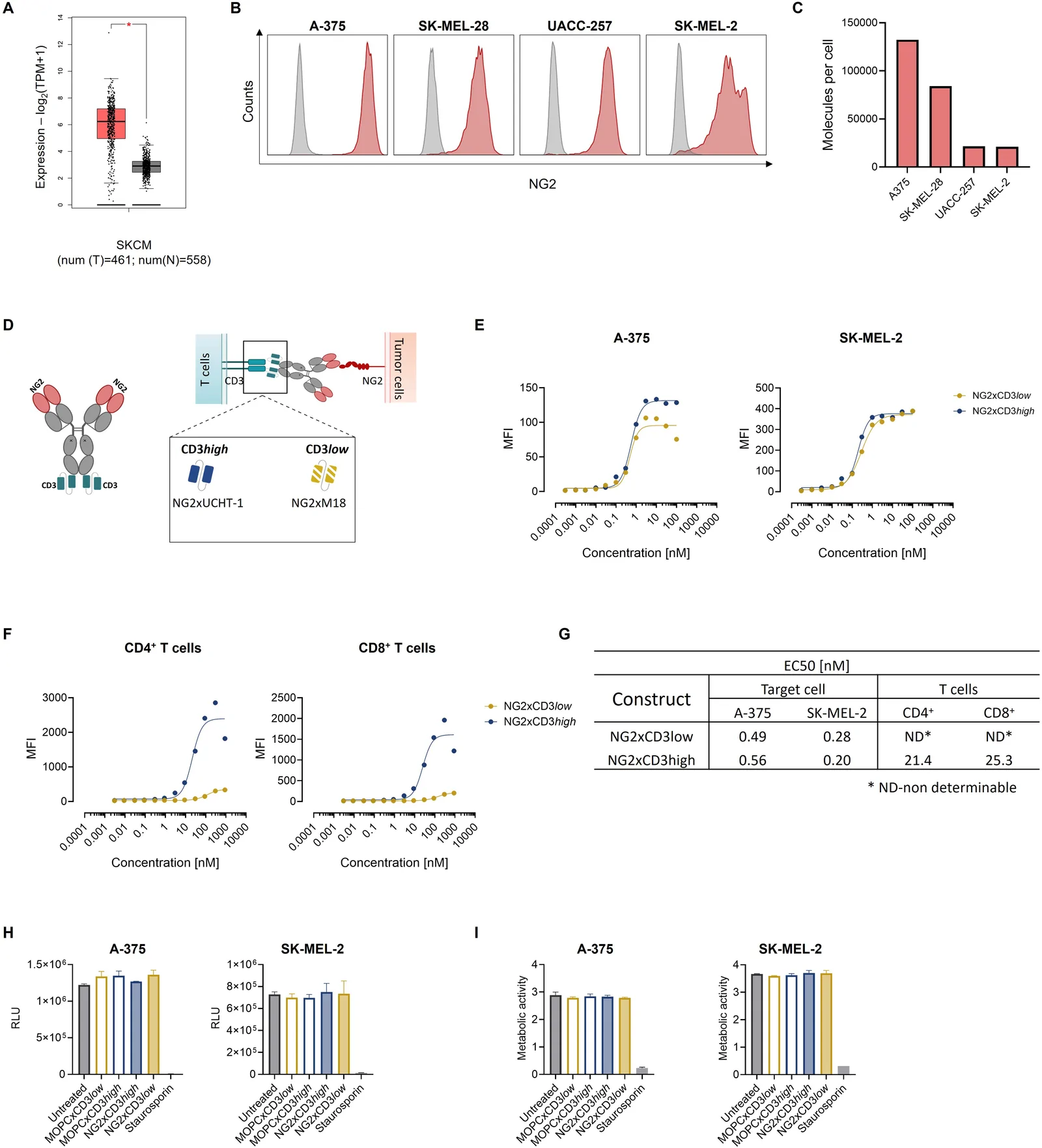
NG2 expression in tumor tissues and generation of NG2xCD3 bsAbs. **A** CSPG4/NG2 mRNA expression in tumor samples (red) and normal skin (grey) was analyzed using TCGA and GTEx datasets via the GEPIA online tool. **B** The indicated melanoma cell lines were stained with the murine 9.2.27 antibody and a PE conjugated anti-mouse IgG secondary antibody, followed by flow cytometric analysis. Histograms show NG2 surface expression (red) for A-375, SK-MEL-2, UACC-257, and SK-MEL-2, compared with the respective mouse isotype controls (grey). **C** NG2 molecules per cell were quantified in the indicated cell lines using flow cytometry and QIFIKIT-based analysis. **D** Schematic representation of the structure and mechanism of action of the two NG2 bsAbs (NG2xCD3high and NG2xCD3low) in the IgG-scFv format including the introduced mutations in the CD3 scFv. Graphic created with BioRender.com. **E** Binding analysis on tumor cells incubated with increasing concentrations of NG2 bsAbs, followed by staining with an PE-conjugated anti-human IgG antibody. Representative data from n = 3 independent experiments. **F** Binding of the indicated antibodies to CD3-positive CD4+ and CD8+ T-cells within healthy donor PBMCs assessed by flow cytometry. Representative data from n = 2 PBMC donors. **G** Summary of EC₅₀ values calculated using GraphPad Prism. **H** ATP levels of A375 and SK-MEL-2 melanoma cells after 72 h treatment with NG2xCD3high, NG2xCD3low, the corresponding isotype control antibodies, or staurosporine as a positive control were determined using the CellTiter-Glo assay. **I** Metabolic activity of the corresponding cultures was measured using the WST-1 assay. Data are shown as mean ± SD of technical quadruplicates. CSPG4. Chondroitin Sulphate Proteoglycan 4, NG2: neuron–glial antigen-2, TCGA: The Cancer Genome Atlas; GTEx: Genotype-Tissue Expression, bsAb: bispecfic antibodies, PBMCs: peripheral blood mononuclear cells

Analysis of TCGA skin cutaneous melanoma (SKCM) and normal tissue from the GTEx dataset revealed significantly elevated NG2 mRNA expression in melanoma. Among tumor entities with increased NG2 expression, SKCM displayed the highest median NG2 transcript levels, consistent with NG2 being a melanoma-associated antigen (Fig. 1A and Figure S1A). To translate these findings into experimental models, we selected four melanoma cell lines based on NG2 mRNA expression reported in the Human Protein Atlas. Surface NG2 expression was then assessed by flow cytometry using the murine anti-NG2 monoclonal antibody 9.2.27. Histogram analysis using PE-conjugated secondary antibody showed detectable NG2 on the cell surface of all four tested cell lines (Fig. 1B). Quantitative flow cytometry using the QIFIKIT estimated the antigen density, with A-375 cells expressing the highest at 132,457 NG2 molecules per cell and SK-MEL-2 cells the lowest at 21,228 molecules per cell among the tested cell lines (Fig. 1C). These two lines were therefore chosen for subsequent functional studies to reflect the impact of high versus lower antigen density. NG2 was not detectable on PBMCs from healthy donors (Figure S1B).

NG2xCD3 bsAbs were generated by humanizing the variable domains of the murine anti-NG2 antibody 9.2.27 and cloning them into our established IgG-scFV (IgGsc) format [26]. For the T-cell-engaging part, two different CD3 binders were used. The CD3*high* molecule contained a single-chain CD3 binder derived from the humanized UCHT1 antibody used in our PSMAxCD3 and FLT3xCD3 formats that presently undergo clinical evaluation (NCT04104607, NCT04496674, and NCT05143996). The CD3*low* variant was based on the same UCHT1-derived sequence but included four mutations in CDR-H2 and one in framework H3, which were introduced to reduce CD3 affinity, as reported for the B7H3xCD3 construct (NCT05999396) (Fig. 1D) [27]. The resulting bsAbs were purified, and assessed by SDS-PAGE and SEC. Both analyses confirmed the expected molecular integrity and overall homogeneity of the constructs (Figure S1C and S1D).

Binding titrations on NG2-positive melanoma cells showed that all NG2xCD3 variants retained comparable NG2-dependent binding, with very similar apparent EC₅₀ values (Fig. 1E and G, S1E and S1F). This demonstrates that adjusting CD3-affinity did not affect recognition of the tumor antigen. As expected, dose titrations on primary T-cells clearly separated the two CD3 variants: the CD3*high* construct displayed EC₅₀ values of approximately 20nM, whereas binding of the CD3*low* bsAb was detectable only at much higher concentrations. (Figure 1F and G). Given the established role of NG2 in melanoma biology, we next investigated whether engagement of NG2 by our bispecific antibodies directly affects melanoma cells in the absence of T cells. Neither NG2xCD3*high* nor NG2xCD3*low* affected cell viability (Fig. 1H) or metabolic activity (Fig. 1I) in A375 or SK-MEL-2 cells compared with untreated and isotype-treated controls. In contrast, the positive control staurosporine induced the expected marked reduction in both assays. These findings suggest that the antitumor activity of NG2xCD3 bsAbs is primarily mediated through T-cell recruitment rather than direct effects on melanoma cells.

### NG2xCD3 bsAbs form artificial immune synapses inducing T-cell activation

**Fig. 2:**
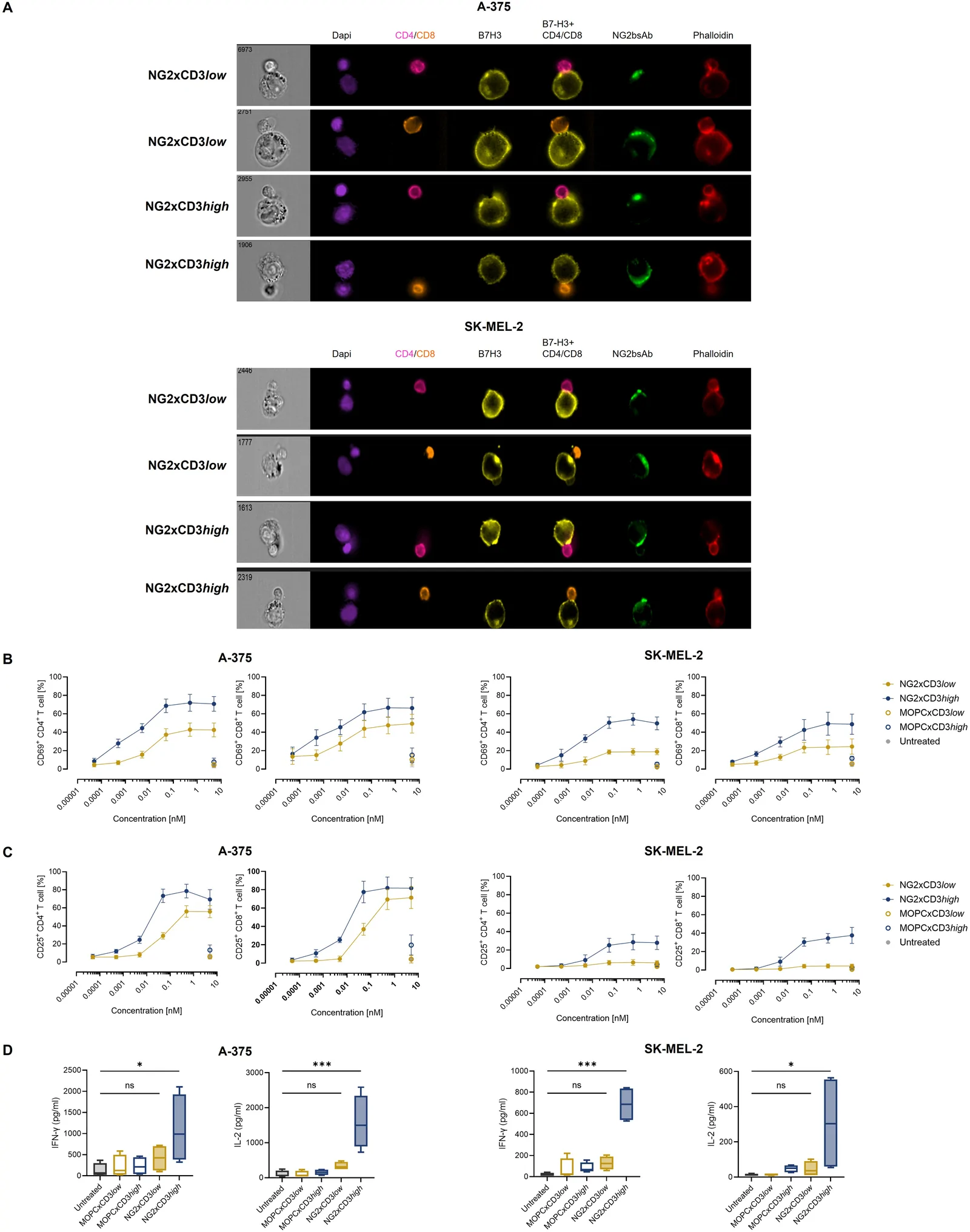
Induction of T-cell localization, early activation, and cytokine secretion by NG2xCD3 bsAbs. PBMCs from healthy donors were incubated with indicated tumor cell lines (Top panel: A-375 and bottom panel: SK-MEL-2) at an E: T ratio of 5:1 in the presence or absence of the two NG2 bsAbs or MOPCxCD3 controls (5 nM). **A** The colocalization and formation of immune synapses was analyzed after 1.5 h of incubation using amnis ImageStream mk II. The formation of the immune synapse between T-cell and target cell doublets was analyzed by measuring colocalization in the overlapping region of polarized phalloidin. **B**-**D** Functional characterization of T cell activation and cytokine release upon indicated treatments. For all panels, left: A-375 and right: SK-MEL-2. **B** CD4⁺ and CD8⁺ T-cell activation quantified by flow cytometry after 24 h of culture with the indicated tumor cell lines. **C** Late T-cell activation after 72 h of co-culture with the indicated tumor cell lines. Isotype controls are represented by open circles; untreated controls by grey dots. Data represent combined results from four PBMC donors (n = 4). **D** IL-2 and IFN-γ levels in culture supernatants measured after 24 h using a LEGENDplex assay. Data represent combined results from four PBMC donors (n = 4). Data are shown as mean ± SD. P values were calculated using one-way ANOVA followed by Dunnett’s multiple comparisons test for normally distributed (Gaussian) data, or Kruskal-Wallis test followed by Dunn’s multiple comparisons test for non-normally distributed data. Significance is indicated as ns (not significant), *p < 0.05, ****p < 0.0001. E: T: effector to target, bsAb: bispecific antibodies, PBMCs: peripheral blood mononuclear cells

To test how the NG2xCD3 bsAbs link NG2‑positive tumor cells with T-cells and thereby form an immune synapse that initiates T‑cell activation, healthy donor PBMCs were cultured with A‑375 (NG2-high) or SK‑MEL‑2 (NG2-low) cells in the presence of NG2xCD3 bsAbs, and cell conjugates were analyzed by imaging flow cytometry. Colocalization of tumor cells and T-cells was assessed by gating on doublets, NG2xCD3 binding was detected using a fluorescently labeled anti‑human mAb, and immune synapse formation was visualized by phalloidin staining of F‑actin at the T-cell-tumor cell interface. These analyses showed accumulation of NG2xCD3 at the contact zone in conjugates for both NG2xCD3 variants and with both melanoma cell lines (Fig. 2A and S2).

Early T‑cell activation was assessed by flow cytometry for CD69 expression on CD4^+^ and CD8^+^ T-cells after 24 h, revealing a clear dose‑dependent increase for NG2xCD3*high*. In contrast, NG2xCD3*low* induced measurable activation only in cultures with NG2‑high A‑375 cells, but not with SK‑MEL‑2, indicating that lower CD3 affinity requires higher antigen density for efficient T-cell activation (Fig. 2B). Similar trends were observed for the late activation marker CD25 (Fig. 2C). Importantly, neither NG2xCD3*high* nor NG2xCD3*low* induced CD25 upregulation in co-cultures with the NG2-negative melanoma cell line MEL-JUSO, further confirming the target antigen-dependent activity of both bispecific antibodies (Figure S3A, B). Analysis of cytokine release showed that only NG2xCD3*high* robustly induced IL‑2 and IFN‑γ with both melanoma lines (Fig. 2D). In all experiments, control bsAbs with irrelevant tumor specificity (MOPCxCD3*low* and MOPCxCD3*high* and) did neither induce T‑cell activation nor cytokine release, confirming strictly NG2‑dependent activity of the NG2xCD3 antibodies.

### NG2xCD3 bsAbs induce T-cell proliferation and memory formation

**Fig. 3:**
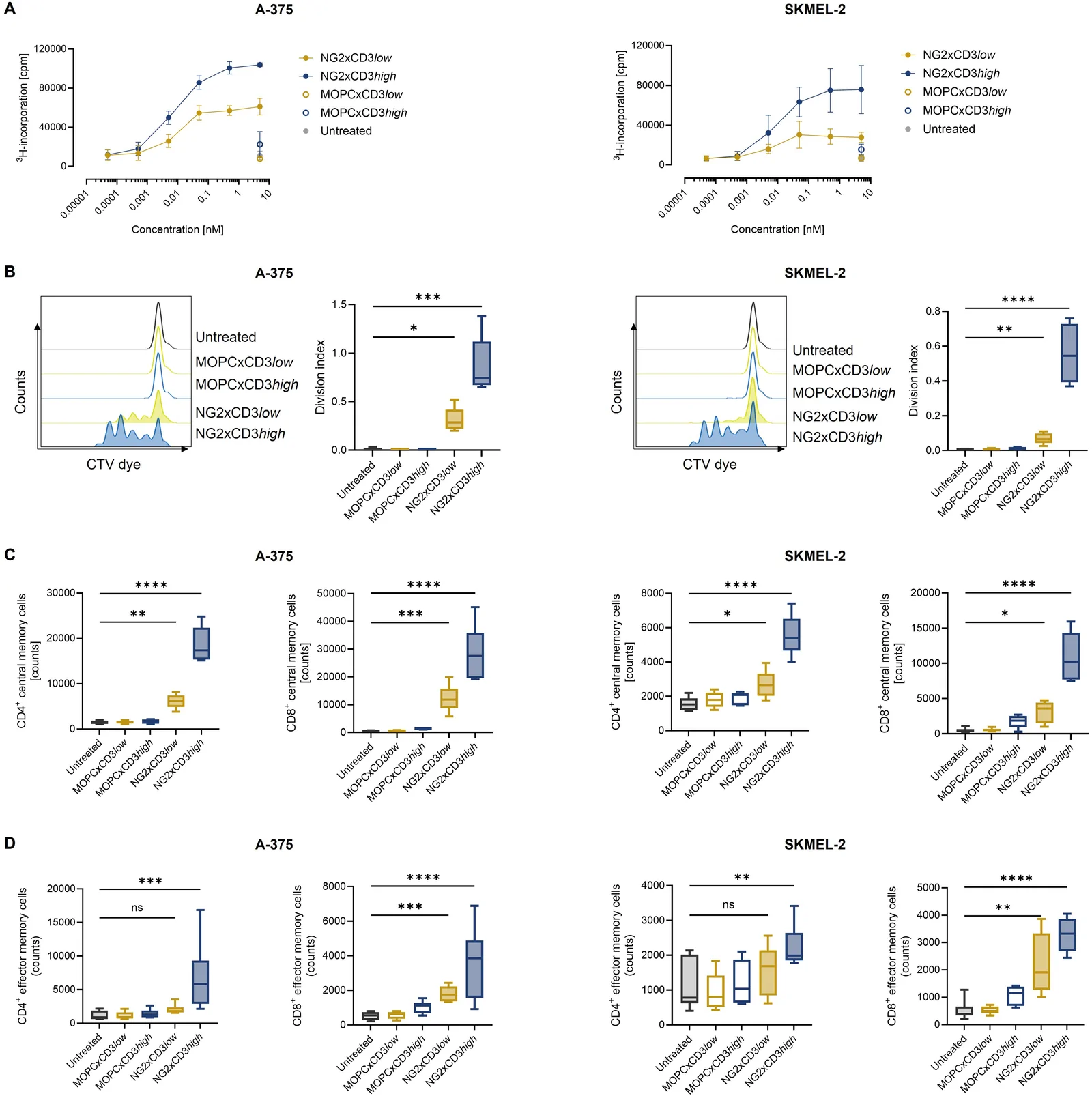
Induction of T-cell proliferation and memory T-cell formation by NG2 bsAbs. **A** PBMCs were cultured with irradiated melanoma cells (Left: A-375; Right: SK-MEL-2) at E: T = 10:1 in the presence of the indicated NG2xCD3 bsAbs or corresponding controls at the shown concentrations for 72 h. T-cell proliferation was quantified by 3H-thymidine incorporation. Data represent four independent PBMC donors (n = 4, data shown as mean ± SD). **B** For flow cytometry-based proliferation analysis, PBMCs were labeled with CellTrace™ Violet and cultured with the indicated tumor cell lines (left: A-375; right: SK-MEL-2) (E: T = 10:1) in the presence of NG2 bsAbs (5 nM) or isotype control. After 72 h, fresh tumor cells and the respective treatments were added for an additional 24 h. On day 4, CTV dilution was analyzed by flow cytometry. Within each cell line, left panels show representative histograms from one donor, analyzed in FlowJo and right panels summarize the division index from four donors (n = 4, mean ± SD). **C**, **D** On day 6, CD4+ and CD8+ T-cell subsets were analyzed to assess memory formation. For memory profiling, unlabeled PBMCs were cultured with tumor cells (left: A-375; right: SK-MEL-2) under the conditions described above. After 72 h, fresh antibodies and fresh target cells were added, and cultures were maintained for an additional 72 h. Memory subsets were defined as follows: effector (CD62L− CD45RO−), naïve (CD62L+ CD45RO−), effector memory (CD62L− CD45RO+), and central memory (CD62L+ CD45RO+). P values were calculated using one-way ANOVA followed by Dunnett’s multiple comparisons test for normally distributed (Gaussian) data, or Kruskal-Wallis test followed by Dunn’s multiple comparisons test for non-normally distributed data. Significance is indicated as ns (not significant), *p < 0.05, ****p < 0.0001. E: T: effector to target bsAbs: bispecific antibodies, CTV: CellTrace™ Violet; PBMCs: peripheral blood mononuclear cells

Given the observed differences in activation and cytokine production, we next investigated whether NG2xCD3*high* and NG2xCD3*low* also differed in their ability to drive sustained T-cell proliferation and the formation of defined memory subsets. In 72 h cultures of healthy donor PBMCs with A‑375 or SK‑MEL‑2 cells, analysis of ³H-thymidine incorporation revealed that NG2xCD3*high* induced pronounced T‑cell proliferation with both cell lines, whereas NG2xCD3*low* induced relevant proliferation only with NG2‑high A‑375, and to a lower extent than NG2xCD3*high*, which is in line with the data on T-cell activation (Fig. 3A). Dye‑based proliferation assays yielded similar proliferation curves and division indices, confirming these results (Fig. 3B, S4A).

To test whether a longer culture period could compensate for lower CD3-affinity, experiments were extended to 6 days and T‑cell subsets were analyzed by flow cytometry. Under these conditions, NG2xCD3*high* induced a marked expansion of central and effector memory CD4^+^ and CD8^+^ T-cells. NG2xCD3*low* also induced substantial memory populations, with increasing frequencies over time. These findings suggest that even the lower‑affinity CD3 binder can support effective T‑cell proliferation and memory development upon prolonged target engagement with both NG2‑high and NG2‑lower expressing tumor cell lines. In line, both NG2xCD3*high* and NG2xCD3*low* were associated with a marked decrease in the naïve T‑cell compartment, supporting the notion that NG2xCD3 engagement drives differentiation of naïve T-cells into memory subsets (Fig. 3C and D and S4B). To further characterize the long-term functional status of NG2xCD3-activated T cells, the expression of the inhibitory receptors PD-1, TIM-3, and LAG-3 was analyzed after 6 days of co-culture. While both NG2xCD3 constructs increased PD-1 expression predominantly on CD4⁺ T cells, co-expression of multiple inhibitory receptors remained limited, with a significant increase in PD-1⁺TIM-3⁺LAG-3⁺ CD4⁺ T cells observed only after NG2xCD3high treatment in A375 co-cultures. CD8⁺ T cells showed only minor changes in inhibitory receptor expression (Figure S5).

### NG2xCD3 bsAbs mediate cytotoxicity in short- and long-term assay

**Fig. 4:**
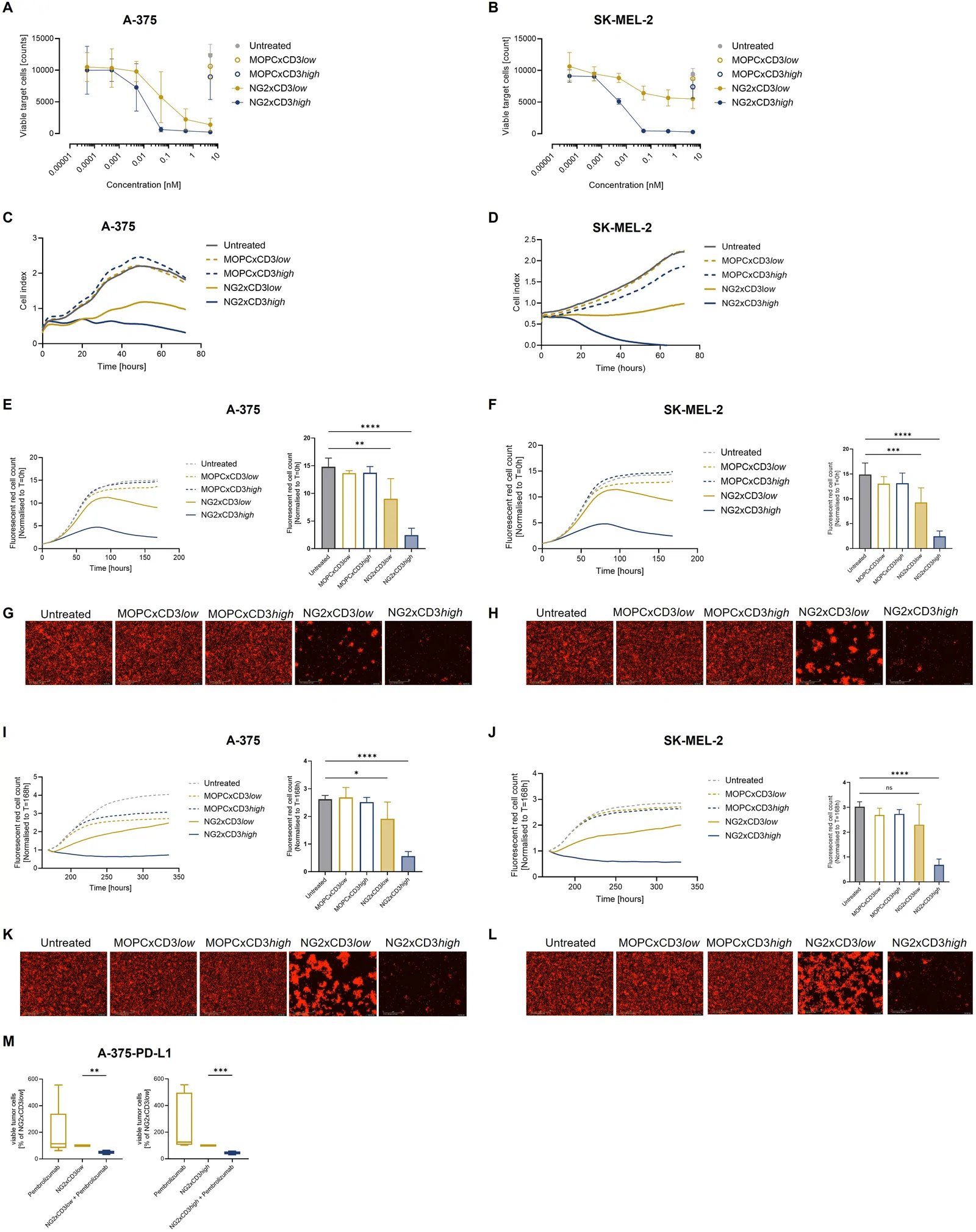
NG2 bsAbs induce tumor cell cytotoxicity. PBMCs were cultured with the A-375 (left) or SK-MEL-2 (right) (E: T = 10:1) in the presence of indicated NG2xCD3 bsAbs or the corresponding isotype controls. **A**, **B** Tumor cell lysis after 72 h was quantified by flow cytometry. Data are shown as mean ± SD (n = 4). **C**, **D** Real-time cytotoxicity was assessed by impedance-based measurements using the xCELLigence system over 72 h at 5 nM antibody bsAbs concentration. Mean cell index values from experiments using n = 4 healthy PBMC donors are shown. **E**–**H** Long-term killing was monitored over 7 days using live-cell imaging (IncuCyte) at 5nM bsAb. Tumor cells were labelled with NucLight Red and quantified by automated image analysis using the red object mask generated by the IncuCyte software. **E**, **F** Left panels show killing kinetics as mean fluorescent red cell count normalised to T = 0 across n = 4 independent donors. (5 nM bsAb) Right panel shows tumor cell counts extracted at day 6 and plotted as mean ± SD in bar graphs to enable statistical comparison. **G**, **H** Representative Incucyte images of tumor cells on day 6 under the indicated conditions, with red objects corresponding to Nuclight red labelled cells in the red fluorescence channel, as defined by the quantified red object mask generated by the Incucyte analysis software. **I**–**L** Extended killing assay in which fresh tumor cells and antibodies were replenished after 7 days (t=168 h), followed by an additional 7 days of cytotoxicity monitoring. **M** PBMCs were co-cultured with CTV labeled A375-PD-L1 cells (E: T = 5:1) in the presence of the indicated NG2xCD3 bsAbs with or without pembrolizumab. After 72 h, fresh target cells together with fresh antibodies and pembrolizumab were added, and cultures were maintained for an additional 72 h. Tumor cell lysis was quantified by flow cytometry after 6 days. Data are presented as viable target cells. (mean ± SD, n = 4). P values were calculated using one-way ANOVA followed by Dunnett’s multiple comparisons test for normally distributed (Gaussian) data, or Kruskal-Wallis test followed by Dunn’s multiple comparisons test for non-normally distributed data. Significance is indicated as ns (not significant), *p < 0.05, ****p < 0.0001. E: T: effector to target, CTV: CellTrace™ Violet; PBMCs: peripheral blood mononuclear cells

To determine whether the observed T‑cell activation and proliferation translated into effective target cell killing, cytotoxicity was assessed over multiple time points. 72 h flow cytometry-based assays quantifying fluorescence labeled melanoma target cells showed a pronounced dose‑dependent killing of both A‑375 and SK‑MEL‑2. With NG2-high A-375 cells, NG2xCD3*high* and NG2xCD3*low* achieved comparable lysis at high concentrations, whereas with SK‑MEL‑2, NG2xCD3*high* exhibited clearly superior cytotoxicity across all tested concentrations (Fig. 4A and B). In contrast, neither NG2xCD3high nor NG2xCD3low induced significant killing of the NG2-negative melanoma cell line MEL-JUSO, further demonstrating the strict target antigen dependence of tumor cell lysis (Figure S3C). Similar kinetics were obtained in an impedance‑based real‑time cytotoxicity assay using the xCELLigence system, which continuously monitored the decline in cell index as a readout of adherent tumor cell loss over approximately 72 h (Fig. 4C and D).

Cytotoxic effects over longer periods of time were evaluated by live‑cell imaging of fluorescence-labeled target cells using Incucyte‑based assays. Here, co‑cultures were maintained for 7 days (Fig. 4E-H), after which fresh target cells and antibodies were added to introduce a re-challenge, and killing was monitored for an additional 7 days (Fig. 4I-L). NG2xCD3*high* induced robust and sustained tumor cell killing over the entire observation period and remained the most potent following rechallenge. NG2xCD3*low* also mediated detectable cytotoxicity against NG2-high A-375 cells, but its activity was consistently lower compared with NG2xCD3*high*, particularly after tumor rechallenge. As expected, the MOPCxCD3 control constructs did not induce relevant tumor cell killing. Given the clinical relevance of PD-1 blockade in melanoma, we next investigated whether NG2xCD3-mediated tumor cell killing could be further enhanced by pembrolizumab. While the addition of pembrolizumab did not significantly increase cytotoxicity during the initial 72 h co-culture (Figure S6), a significant enhancement of NG2xCD3-mediated tumor cell killing was observed following target cell rechallenge after 6 days of culture (Fig. 4M). These findings indicate that the beneficial effects of PD-1 blockade become apparent during prolonged co-culture and repeated target cell encounter, potentially reflecting a more prominent role of PD-1/PD-L1-mediated inhibitory signalling over time.

Together, these data demonstrate that NG2-directed T-cell engagement translates into effective melanoma cell killing in both short- and long-term settings. Moreover, the superior and sustained activity of NG2xCD3high, particularly under rechallenge conditions, identifies this format as the more potent construct for durable anti-tumor responses.

## Discussion

Immunotherapy with CD3-recruiting bsAbs has transformed the treatment landscape particularly in hematological malignancies, and their efficacy in solid tumors was long hampered by target specificity, heterogenous antigen expression and restricted T-cell access. However, emerging clinical data now indicate that CD3 bispecifics can also induce meaningful and durable responses in certain solid tumors [34–36]. Recent preclinical and early clinical studies have highlighted the need for careful optimization of both the target and the CD3 binder, as there is no ‘universal’ CD3 binder that is optimal across indications [37–39]. Instead, parameters including CD3 epitope, binding affinity, valency, and overall molecular geometry must be tuned for each target antigen to achieve potent tumor cell killing while minimizing on‑target, off‑tumor toxicity [27, 40, 41].

NG2 has emerged as an attractive target for bsAbs because of its high and relatively tumor-restricted expression in aggressive melanoma and other malignancies. In contrast to many clinically explored solid tumor antigens such as CEA, EpCAM, HER2 or PSMA, NG2 is an exceptionally large, heavily glycosylated transmembrane proteoglycan the expression of which is strongly enriched in melanomas, selected sarcomas and gliomas, as well as tumor‑associated pericytes. The latter may influence both antigen accessibility and the geometry of T-cell engagement [42]. There are previous reports of NG2 targeting bispecific antibodies establishing the possibility of NG2-dependent T-cell redirection [43]. Notably, Torisu-Itakura et al. explored NG2 in a BiTE format targeting CD3, demonstrating dose-dependent lysis of NG2 expressing melanoma cell lines, with both healthy-donor and patient-derived T cells [22]. Recent immunohistochemical analyses further support the translational relevance of NG2 as a therapeutic target by demonstrating protein expression in approximately two thirds of both primary and metastatic melanoma specimens, suggesting that NG2 expression is largely maintained during disease progression. Expression was observed across the major melanoma subtypes, although with varying frequencies, highlighting both the broad applicability of NG2-directed therapies and the importance of patient stratification based on target expression. While the present study was intentionally performed in well-characterized melanoma cell lines to enable systematic functional comparison of the bispecific constructs, validation in patient-derived melanoma models and ex vivo tumor specimens will represent an important next step to account for interpatient heterogeneity and the complexity of the tumor microenvironment [44].

Acquired resistance to immune checkpoint inhibitors remains a major clinical challenge in advanced melanoma. Recent clinical analyses suggest that NG2 expression is largely maintained following treatment with immune checkpoint inhibitors and BRAF/MEK inhibitors, supporting its continued suitability as a therapeutic target even after disease progression [45]. This observation is consistent with recent mechanistic studies demonstrating that melanoma cells undergoing immune-driven dedifferentiation retain a NG2-positive, neural crest-like phenotype [46]. As NG2xCD3 bispecific antibodies redirect T cells through direct CD3 engagement independently of peptide-MHC recognition, they may provide a complementary therapeutic strategy to target NG2-positive melanoma cells that have escaped conventional T-cell recognition. Moreover, by establishing a new mechanism of T-cell activation, NG2-directed T-cell engagers could potentially restore susceptibility to checkpoint inhibition in combination approaches, although this hypothesis requires validation in dedicated preclinical models of ICI-resistant melanoma.

Here we report on the generation and comprehensive characterization of a tetravalent Fc-silenced IgG-ScFv NG2xCD3 bsAbs incorporating the humanised 9.2.27 NG2-binding domain. We systematically compared variants harboring high versus low high affinity CD3 binding arms [26, 27]. Bispecific formats such as BiTEs, as well as other constructs comprising multiple single-chain fragments, are prone to aggregation, which can result in off-target immune activation and cytokine release. In contrast, the full-length IgG-Sc format provides clear pharmacokinetic advantages, including prolonged serum half-life, as well as improved manufacturability and molecular stability [47–50]. 

Importantly, Fc silencing abolishes Fcγ receptor (FcγR) engagement, thereby preventing Fc-mediated, target-independent activation of FcγR-expressing effector cells such as NK cells and myeloid populations. This design feature contributes to enhanced tumor specificity by restricting immune activation to CD3 engagement in the presence of NG2-positive target cells [47, 51]. We further systematically compared high and low affinity CD3 binders within this format across NG2-high and low expressing tumor cell lines using a comprehensive panel of different functional readouts.

Imaging flow cytometry demonstrated that our bsAbs induce the formation of organized immunological synapses for both, low and high CD3 affinities, indicating that the molecular geometry of NG2 is sufficient to bridge the large, glycosylated NG2 ectodomain on tumor cells with CD3 on T-cells and to stabilize productive cell-cell contacts. This is particularly important given that the 9.2.27 antibody acting as target binder in our constructs binds a relatively distal epitope of NG2, which might constrain synapse formation [24, 43].

Immunological synapse formation resulted in robust tumor cell killing at comparatively moderate levels of T-cell activation and cytokine release in the range of what has been reported for other CD3 bsAbs [52, 53]. Thus, targeting NG2 may allow efficient T-cell-mediated elimination of NG2-positive cancer cells.

As prolonged T-cell stimulation may promote the expression of inhibitory receptors and eventually lead to T-cell dysfunction, we further analyzed the phenotype of T cells following extended NG2xCD3-mediated stimulation. While PD-1 expression increased, particularly within the CD4⁺ T-cell compartment, only limited co-expression of additional inhibitory receptors such as TIM-3 and LAG-3 was observed. Together with the sustained cytotoxic activity following target cell rechallenge, these findings suggest that increased PD-1 expression primarily reflects ongoing antigen-driven activation rather than the establishment of a broader exhaustion phenotype. Interestingly, the addition of pembrolizumab significantly enhanced NG2xCD3-mediated tumor cell killing only after prolonged co-culture and repeated target cell encounter, whereas no additional effect was observed during the initial 72 h. This observation is consistent with the notion that PD-1/PD-L1-mediated inhibitory signalling becomes increasingly relevant during sustained T-cell activation and supports the rationale for combining NG2-directed T-cell engagers with immune checkpoint blockade.

When directly comparing the two CD3 affinity variants, the UCHT-1 based NG2xCD3high bsAb consistently emerged as the superior construct. It mediated more sustained tumor cell killing in long-term assays, induced stronger cytokine responses, and promoted a more pronounced expansion of central and effector memory T cells than the lower-affinity variant. In contrast, NG2xCD3low remained functional but showed reduced activity, particularly against NG2-low melanoma cells, underscoring the importance of optimizing CD3 affinity for solid-tumor targeting. The observed differences in T-cell subset distribution likely reflect the impact of CD3 signalling strength on T-cell differentiation. While both constructs promoted the transition of naïve T cells into memory populations following prolonged target engagement, the stronger CD3 engagement provided by NG2xCD3high resulted in more pronounced memory differentiation. These findings are consistent with the concept that the magnitude of CD3-mediated signalling shapes T-cell differentiation and highlight the importance of carefully balancing CD3 affinity to achieve durable anti-tumor activity while maintaining functional T-cell responses [54].

## Conclusion

Taken together, these findings position NG2 as a biologically and pharmacologically promising target for T-cell-redirecting therapy and underscore the need to co‑optimize target and CD3 binders within a given bsAb. The tetravalent NG2xCD3 IgG‑scFv constructs described here provide proof‑of‑concept that effective immunological synapse formation and durable cytotoxicity can be achieved when targeting a large, heavily glycosylated solid‑tumor antigen, with a bias toward activity in NG2‑high tumors and when using UCHT-1 based high affinity CD3 binders which favours sustained killing and memory formation. Future work might systematically interrogate NG2 epitope variants, extend evaluation to in vivo models and safety studies, and explore combinations with checkpoint inhibitors or other microenvironment‑modifying agents to fully realize the therapeutic potential of NG2‑directed CD3 bsAbs.

## Supplementary Information

Below is the link to the electronic supplementary material.

Supplementary Material 1

## Data Availability

The data generated in this study are available within the article and its supplementary data files.
