# Supplementary material for: Generation of an NG2 targeting bispecific antibody for the induction of T-cell immunity against melanoma
Source: J Transl Med. 2026 Sep 19;24:1204. doi: 10.1186/s12967-026-09000-5 (PMC13595633; doi:10.1186/s12967-026-09000-5)
Supplement: Supplementary file 1 [file 12967_2026_9000_MOESM1_ESM.docx]

**Supplementary Figure S1. NG2 expression in various cancers and healthy donor PBMCs and biochemical characterization of NG2xCD3 bsAbs.** **A** NG2 expression levels across cancer types were analyzed using the GEPIA tool, highlighting tumor entities with high NG2 expression. **B** Binding of NG2 was further assessed in the presence (grey-filled) or absence (black-dotted line) of the murine NG2 antibody-9.2.27 across PBMCs from five donors **C** Biochemical characterization of the generated NG2xCD3 bsAbs by SDS-PAGE. **D** Size-exclusion chromatography (SEC) analysis of the NG2xCD3 bsAbs. **E** Binding analysis of the indicated antibodies using increasing concentrations, followed by detection with a PE-conjugated secondary antibody. **F** Summary of EC₅₀ values. NG2: neuron–glial antigen-2. PBMCs: peripheral blood mononuclear cells. SDS-PAGE: sodium dodecyl sulfate–polyacrylamide gel electrophoresis. SEC: Size-exclusion chromatography.

**Supplementary Figure S2. T-cell localization on melanoma cells expressing B7-H3. A** Surface expression of B7-H3 on the two melanoma cell lines used in the study. B7-H3 served as a marker to identify target cells in both ImageStream and flow-cytometric analyses. **B** T cells from healthy donors were incubated with indicated tumor cell lines at an E: T ratio of 5:1 in the presence of the two NG2 bsAbs (5 nM). The colocalization and formation of immune synapses was analyzed after two hours of incubation using amnis ImageStream mk II. The formation of the immune synapse between T cell and target cell doublets was analyzed by measuring colocalization in the overlapping region of polarized phalloidin. bsAbs: Bispecific antibodies. E:T: effector to target

**Supplementary Figure S3. NG2xCD3 bsAbs do not induce target antigen-independent T-cell activation or tumor cell killing.** **A** MEL-JUSO cells were stained with the murine 9.2.27 antibody followed by a PE-conjugated anti-mouse IgG secondary antibody and analyzed by flow cytometry. Histograms show NG2 surface expression (red) compared with the respective mouse isotype control (grey). **B-C** Healthy donor PBMCs were co-cultured with the NG2-negative melanoma cell line MEL-JUSO at an effector-to-target (E:T) ratio of 5:1 in the presence of NG2xCD3high, NG2xCD3low, or the corresponding MOPCxCD3 control antibodies (5 nM). **B** CD25 expression on CD4+ and CD8+ T cells was quantified by flow cytometry after 72 h of co-culture. Isotype controls are shown as open circles and untreated controls as grey circles. Data represent pooled results from four healthy PBMC donors (n=6). **C** Tumor cell lysis after 72 h was quantified by flow cytometry. Data are shown as mean ± SD (n=6).

**Supplementary Figure S4**. **Proliferation profiles of additional donors and reduction of naïve T-cell populations**. **A**. Proliferation profiles from additional PBMC donors following co-culture with NG2 bsAbs, as assessed by CellTrace^TM^ Violet dilution: **B**. Reduction of naïve T-cell frequencies, defined as CD62L⁺ single-positive cells, following treatment under the same conditions. Data are shown as mean ± SD. P values were calculated using one-way ANOVA followed by Dunnett’s multiple comparisons test for normally distributed (Gaussian) data, or Kruskal-Wallis test followed by Dunn’s multiple comparisons test for non-normally distributed data. Significance is indicated as ns (not significant), *p < 0.05, ****p < 0.0001.

**Supplementary Figure S5. Analysis of exhaustion-associated T-cell phenotypes following prolonged NG2xCD3-mediated stimulation.**

PBMCs were co-cultured with the indicated melanoma cells at an effector-to-target (E:T) ratio of 5:1 in the presence of the indicated NG2xCD3 bsAbs or the corresponding isotype controls. After 72 h, fresh target cells and antibodies were added, and cultures were maintained for an additional 72 h. On day 6, CD4⁺ and CD8⁺ T cells were analyzed by flow cytometry for the expression of the inhibitory receptors PD-1, TIM-3, and LAG-3. T-cell subsets were categorized according to the expression of inhibitory receptors as **A**: PD-1⁺ cells, **B:** PD-1⁺TIM-3⁺ double-positive cells, and **C:** PD-1⁺TIM-3⁺LAG-3⁺ triple-positive cells . Data are shown as mean ± SD (n=6). P values were calculated using one-way ANOVA followed by Dunnett's multiple comparisons test for normally distributed (Gaussian) data, or Kruskal-Wallis test followed by Dunn's multiple comparisons test for non-normally distributed data. Significance is indicated as ns (not significant), *p < 0.05, ***p < 0.0001. E:T, effector-to-target ratio; bsAbs, bispecific antibodies; PBMCs, peripheral blood mononuclear cells.

**Supplementary Figure S6. Effect of PD-1 blockade on NG2xCD3-mediated tumor cell killing during initial co-culture.** PBMCs were cultured with CTV labeled A375-PD-L1 cells (E:T = 5:1) in the presence or absence of of NG2xCD3*low*bsAb with or without pembrolizumab and **B** NG2xCD3*high* bsAb with or without pembrolizumab. Tumor cell lysis after 72 h was quantified by flow cytometry. . Data are shown as mean ± SD (n=4). P values were calculated using one-way ANOVA followed by Dunnett’s multiple comparisons test for normally distributed (Gaussian) data, or Kruskal-Wallis test followed by Dunn’s multiple comparisons test for non-normally distributed data. Significance is indicated as ns (not significant), *p < 0.05, ****p < 0.0001. E:T: effector to target bsAbs: bispecific antibodies, CTV: CellTrace™ Violet; PBMCs: peripheral blood mononuclear cells.
